# A Novel Two-Chamber Setup for Containment Investigations with Special Focus on the Dustiness of Pharmaceutical Powders Depending on the Airflow

**DOI:** 10.3390/pharmaceutics14112387

**Published:** 2022-11-05

**Authors:** Steffen Wirth, Martin Schöler, Jonas Brügmann, Claudia S. Leopold

**Affiliations:** 1 Department of Pharmaceutical Technology, University of Hamburg, Bundesstr. 45, D-20146 Hamburg, Germany; 2 Fette Compacting GmbH, Grabauer Straße 24, D-21493 Schwarzenbek, Germany

**Keywords:** containment, two-chamber setup, HPAPI, dustiness, dust emission, dust transfer, evacuation time, smoke, computational fluid dynamics

## Abstract

In the present study, it was shown that a newly developed two-chamber setup (TCS) for containment investigations consisting of an emission and a detection chamber may serve to predict the dustiness of HPAPIs in a sealed system at different flow conditions. These flow conditions include the plain diffusive transport and the diffusive transport with the oppositely directed convective flow of airborne particles of the safe surrogate substance acetaminophen (ACAM). A linear correlation was found between an atomized amount of up to 400 mg of ACAM and the resulting dust emissions. The dust emission was reduced significantly by an oppositely directed convective flow. The results from the examinations, using either atomized ACAM or smoke for the determination of the evacuation time of the detection chamber, indicated that both methods are comparable. Furthermore, computational fluid dynamics (CFD) simulations were performed to determine the evacuation time. A time period of 9 min was sufficient for a reproducible evacuation and a reliable detection of most airborne ACAM particles within the detection chamber. CFD simulations were also carried out to simulate the air velocity resulting from various pressure differences and to visualize the flow of the airborne particles within the detection chamber.

## 1. Introduction

In recent years, the number of highly active pharmaceutical ingredients (HPAPIs) has significantly increased, resulting in a high interest of pharmaceutical companies in the safe handling of these hazardous compounds [1,2,3]. For example, in oncological therapy, HPAPIs are commonly used as cytostatics or peptides. Hormones and antibiotics are other examples of high potency compounds with a high potential for hazard exposure to the personnel present in the intermediate environment. Therefore, measures must be taken regarding the technical design of the facilities and the equipment for handling HPAPIs [3,4,5].

The potential hazard exposures may lead to a health risk for the operators, as well as to a contamination of the outdoor environment and to cross-contaminations during manufacturing or other production processes within the facility. Therefore, the generation of dust, gases, or vapors is an important issue in the processing of dosage forms containing HPAPIs. The application of suitable containment equipment and, in some cases, additional personal protective equipment, is essential to ensure safe handling [6,7]. To guarantee the safe manufacture of dosage forms containing HPAPIs, special production setups are applied which are operated at a negative pressure to reduce the risk of potential airborne transfer to the immediate environment. In addition, barrier isolators, special transfer and valve systems, and high performance filters are further governance methods to reduce the risk of hazards [3,8].

Generally, the dustiness of formulations containing HPAPIs is a major problem concerning potential exposure. Thus, the minimization or elimination of any potential exposure is a common safety measure in the production of dosage forms containing HPAPIs [3,9,10].

Dustiness is defined as the propensity of a material to generate airborne particles during its handling [11]. Various processes in pharmaceutical manufacturing, such as processing, milling, grinding, and mixing, are associated with the generation of dust and, consequently, dustiness is a major risk of potential hazard exposure. Surprisingly, the number of studies on the dustiness of pharmaceutical powders is rather low, compared to that on the dustiness of non-pharmaceutical powders. The dustiness of a pharmaceutical powder is influenced by many factors, especially by the physicochemical and mechanical properties of the material and the method of dust determination. Therefore, the measurement of the dustiness of pharmaceutical powders is of particular importance for the assessment of the hazard exposure in pharmaceutical facilities [9,12,13,14,15,16]. For this reason, various systems and methods were developed to measure the dustiness of solid substances in a reproducible manner. Most of these systems are not designed for the measurements of pharmaceutical powders because the required substance quantities for these measurements are relatively high. Accordingly, because of the typically scarcely available quantities of HPAPIs, the measurements are limited and relatively expensive. The compounds used in the pharmaceutical production are exposed to a wide variety of stresses. Thus, no single test system may reproduce all conditions during handling [17,18,19,20,21,22,23].

The atomization of powders results in an airborne particle distribution and is achieved by the application of energy. Therefore, atomization may be obtained by using a mechanical stimulus or by the dispersion of the pharmaceutical powder in the air. The application of too much energy may result in a fragmentation of single particles and possibly, in a greater dustiness. Each method for the determination of the dustiness includes a different technique for atomization. Thus, a comparability of the different methods for determination of the dustiness is nearly unimaginable because of the complexity of the different measurement processes. Moreover, an accurate prediction of the dustiness based on material properties such as particle size and density is not possible. However, the particle size distribution, particle shape, bulk density, humidity, and the cohesive and adhesive forces of powder particles have a major influence on the resulting dustiness. A reproducible dust generation requires a standardized method, and thus detailed specifications, regarding test duration, type, intensity of the mechanical stimulus, and the amount of test material are necessary. Therefore, a high standardization with relation to industrial manufacturing processes is essential for suitable dustiness measurements [9,11,19,24,25,26,27,28].

The assessment of containment equipment and the evaluation of systems and devices for dustiness measurements should be performed using safe surrogate substances. These substances, such as lactose, naproxen sodium, mannitol, acetaminophen, insulin, riboflavin, and sucrose, along with additional information regarding such measurements, are listed in the Good Practice Guide of the International Society for Pharmaceutical Engineering (ISPE) [6]. The choice of the surrogates should not only depend on the physicochemical and mechanical substance properties, but also on the conditions of the analytical quantification [29].

The applications of computational fluid dynamics (CFD) are versatile, and it is currently used as an efficient tool in the pharmaceutical industry for various purposes, such as the characterization of dry powder inhalers, the Venturi dustiness tester, and the Heubach Dustmeter [30,31,32,33]. In addition, CFD is commonly applied to assess systems for the measurement of dustiness. In this context, CFD allows a modeling of the aerodynamics within the systems to assess the homogenous distribution of dust and to identify zones in which dust accumulates at the end of the sampling process. Furthermore, these simulations are also appropriate to investigate a possible inhomogeneous or delayed powder atomization [32]. Moreover, CFD is suitable to describe the atomization process of pharmaceutical powders and to assess their flow behavior within the systems [33,34].

The number of investigations regarding the dustiness of pharmaceutical powders is relatively low compared to the dustiness investigations of non-pharmaceutical powders [21,35]. In addition, in the literature, the description of the distribution of airborne particles in a defined environment as a function of various flows is rather vague.

Based on the above-mentioned remarks, further research is needed to expand the knowledge on the influence of the plain diffusive transport and the diffusive transport with an oppositely directed convective flow of pharmaceutical airborne particles on the dustiness of HPAPIs. Therefore, the objective of this work was to develop a novel TCS consisting of an emission and a detection chamber to investigate the dustiness depending on different flow conditions. A further aim of this study was to confirm that the newly developed TCS ensures a reproducible and precise dustiness investigation of pharmaceutical powders. The different parameters of the atomization phase, transport phase, and detection phase may influence the investigation of the TCS. In this context, both the reproducible and precise atomization of small amounts of the surrogate substance ACAM and the accurate determination of the dust emission by an IOM sampler (Institute of Occupational Medicine) are the crucial parameters that may significantly influence the dustiness investigations in terms of the reproducibility and precision of the results.

Modern equipment for the production of dosage forms containing HPAPIs is typically operated with negative pressure to reduce potential hazard exposure [1]. Consequentially, another objective of this study was the generation of an adjustable oppositely directed convective flow from the detection to the emission chamber within the TCS, simulating the negative pressure for reduction of the potential hazard exposure.

## 2. Materials and Methods

### 2.1. Materials

Acetaminophen (ACAM; Caelo, Hilden, Germany), as an industry-accepted safe surrogate substance, was used to measure dustiness [6]. Furthermore, a Dräger Air Flow Tester (Dräger, Lübeck, Germany) was used to generate smoke for flow visualization.

### 2.2. Two-Chamber Setup

#### 2.2.1. Design of the Two-Chamber Setup

**Figure 1:**
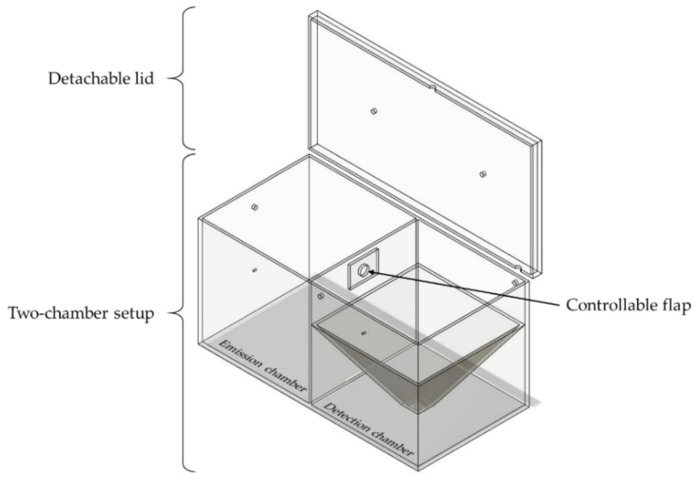
Simplified illustration of the TCS in the open state, consisting of a detachable lid, a controllable flap, and the two connected chambers.

In Figure 1, a simplified illustration of the novel two-chamber setup (TCS) is shown. The setup consists of an emission chamber and a detection chamber, connected by an orifice with a diameter of 25.4 mm. A flap that is controllable via a double acting pneumatic cylinder is installed to separate the chambers, depending on the phase of the measurement course. The emission chamber shows dimensions of 300 mm × 300 mm × 300 mm, resulting in a volume of 27 L. The detection chamber has the same dimensions, but exhibits a smaller volume of approximately 15 L because of the pyramidal construction in the lower section of the chamber.

**Figure 2:**
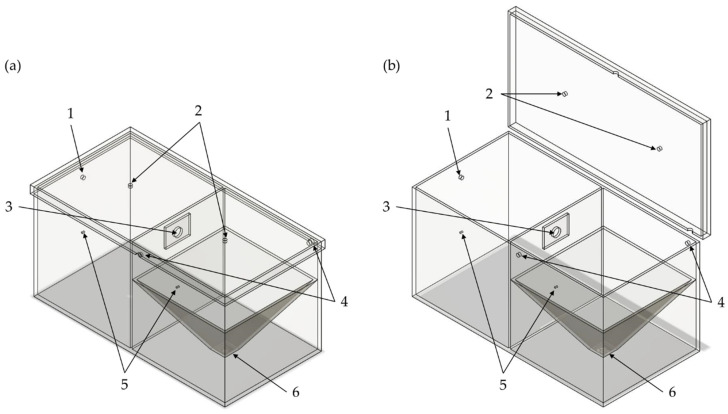
Schematic illustration of the TCS (**a**) in the closed state and (**b**) in the open state. 1: orifice for pressure compensation of the emission chamber; 2: orifices for the double-acting ball valves; 3: orifice for the connection between the emission and the detection chamber; 4: orifices for pressure compensation of the detection chamber; 5: orifices for the differential pressure gauge; 6: orifice for the IOM sampler.

The emission chamber contains an orifice for pressure compensation during the first phase of the measurement course in which ACAM is atomized (Figure 2). The detection chamber comprises two orifices for pressure compensation during the second phase of the measurement course. An IOM sampler is fastened at the bottom of the detection chamber and is connected to the air sampling pump (AirChek ESSENTIAL Pump, SKC, Blendfort Forum, UK). The detachable lid allows for cleaning the TCS after each measurement. The lid contains two orifices for the fastening of two pneumatic ball valves and is attached to the TCS by toggle locks. In addition, a rubber seal is attached to the edges of the chambers to ensure tightness.

The TCS consists of acrylic glass with a wall thickness of 6 mm. Aluminum B type profiles were used to fasten measuring the instruments: a pneumatic cylinder, pneumatic valves and ball valves, as well as a programmable logic controller (PLC). Additionally, a ground cable was attached to the metal parts to minimize electrostatic charge.

The valve control of the TCS is executed by a PLC (Siemens LOGO! 12/24RC, Munich, Germany). The input signal for starting the program of the PLC was generated time-dependently by a single-board microcontroller (Arduino^®^ Uno Rev3, Ivrea, Italy) and amplified with a DC-to-DC converter to a voltage of 12 V. Resulting from the ability of the single-board microcontroller to send a punctual input signal to the PLC, all phases of the measurement course may be controlled as a function of time. Therefore, the air sampling pump and the PLC were synchronized with the server time of a computer. The PLC allows for the generation of various flow conditions within the TCS by controlling the pneumatic ball valves attached to the detachable lid.

**Figure 3:**
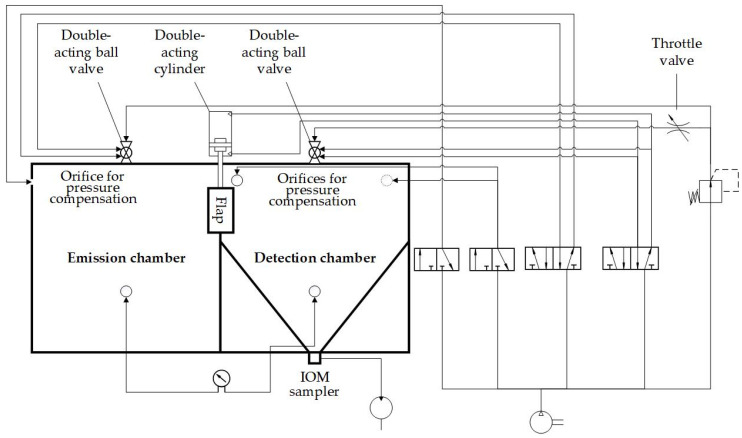
Piping and instrumentation diagram of the TCS.

In Figure 3, the pneumatic valves and devices are shown. The pneumatic ball valve (KH 14 P, Pneumatikatlas, Lübeck, Germany), located above the emission chamber, was used for the atomization of ACAM. Another identical pneumatic ball valve, positioned behind a throttle valve, is located above the detection chamber to generate a flow in the direction of the emission chamber. Both ball valves are controlled pneumatically by 5/2 solenoid valves (VUVS-L25-M52-AD-G14-F8, Festo, Esslingen, Germany). Two 3/2 solenoid valves (VUVS-LK25-M32C-14-B, Festo, Esslingen, Germany) were used to control 3/2 pneumatic valves (SFP4701, YPC, Jeongwang-Dong, South Korea) that manage the pressure compensations. All four solenoid valves are linked to the four outputs of the PLC. All pneumatic components and orifices for pressure compensation are attached to compressed-air tubes (PUN-10X, Festo, Esslingen, Germany). The air sampling pump is connected to the IOM sampler at the bottom of the detection chamber. A digital paddle wheel flow meter (35812, ANALYT-MTC Meßtechnik, Mülheim, Germany) was used to verify the flow rate of the air sampling pump. Additionally, the pressure and temperature difference between the emission and the detection chamber were measured by a differential pressure gauge (testo 400, Testo, Titisee Neustadt, Germany).

#### 2.2.2. Description of the Measurement Course

Three phases may be distinguished during the measurement course of the TCS. The first phase is defined as the atomization phase, in which 100 mg of ACAM were atomized through the ball valve above the emission chamber with an overpressure of 50,000 Pa for a time period of 5 s. The pressure was compensated for by opening the 3/2 pneumatic valve. The controllable flap was closed for the same time period to obtain a homogeneous dispersion of airborne particles and to prevent the transport of these particles from the emission to the detection chamber.

During the second phase, which is called the transport phase of the measurement course, the transport of airborne particles was investigated, depending on the flow conditions. This phase was intended to examine the plain diffusive and the oppositely directed convective transport mechanism of airborne particles in the TCS. In this phase of the measurement course, the controllable flap was opened for 60 s to obtain either a diffusive or a convective flow between both chambers in order to investigate the transport of airborne particles. If there is no pressure difference between the chambers (Δp = 0 Pa), the plain diffusive transport of airborne particles may be investigated. In contrast, a flow from the emission to the detection chamber was generated by an adjustable pressure difference between these chambers to investigate the diffusive flow, reduced by an oppositely directed convective flow, in addition to the plain diffusive transport.

**Figure 4:**
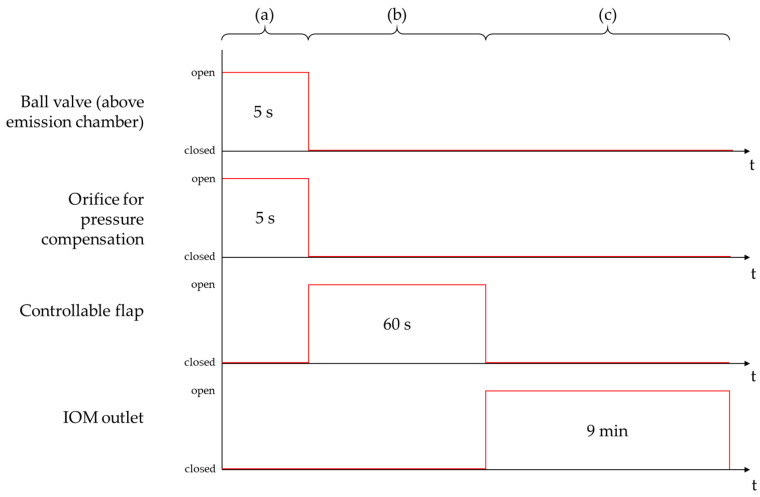
Schematic illustration of the three phases of the measurement course: atomization phase (**a**); transport phase (**b**); detection phase (**c**).

The third phase was defined as the detection phase of the measurement course, during which the dust emission of ACAM within the detection chamber was determined. The controllable flap was closed after 60 s to separate both chambers and to quantify the amount of ACAM transferred from the emission to the detection chamber. The detection chamber was evacuated using the air sampling pump, with a flow rate of 5.0 L/min for 9 min. The pump was connected to the IOM sampler located at the bottom of the detection chamber, which contained a glass microfiber filter (1820–025, Whatman, Little Chalfont, UK), as recommended by the ISPE [6]. In Figure 4, the three phases of the measurement course are shown schematically.

After 9 min, the filter containing the collected dust was transferred to an iodine flask. A moistened glass microfiber filter was used to swab the inner part of the IOM sampler and was then transferred to the same iodine flask. The total collected ACAM was extracted using the mobile phase of the HPLC assay and subsequently quantified (see Section 2.4.).

#### 2.2.3. Investigation of the Atomization Process during the Atomization Phase

This investigation was performed to ensure that small amounts of pharmaceutical powders may be reproducibly atomized in the TCS. The atomization of the surrogate powder ACAM within the emission chamber of the TCS was caused by an overpressure of 50,000 Pa. To examine the influence of the amount of ACAM on the extent of dust emission, the amounts of atomized ACAM were predefined to cover a range between 50 mg and 500 mg, divided into 50 mg portions. The dust emission was measured by evacuating the detection chamber through the filter-containing IOM sampler using the air sampling pump under the above-mentioned conditions. Thereafter, the glass microfiber filters containing the total collected ACAM were analyzed by HPLC. All measurements were performed in sextuplicate.

#### 2.2.4. Investigation of the Dustiness during the Transport Phase in Dependence of the Airflow

In the second phase of the measurement course, the transport of the atomized ACAM between both chambers resulting from various pressure differences between 0 and 4 Pa was investigated. For this purpose, computational fluid dynamics (CFD) simulations and the atomization of ACAM were performed to assess the effect of plain diffusive transport and diffusive transport with an oppositely directed convective flow of airborne particles.

CFD was performed to simulate the air velocity at the orifice between the emission and the detection chamber for the determination of the average air velocity. The simulations were carried out with the SimScale CFD simulator (SimScale, Munich, Germany) using the k-omega shear stress transport turbulence model (k-ω-SST). The initial conditions of the CFD simulations are shown in Table 1.

An overview of the airflow properties chosen for the simulations is presented in Table 2. The boundary conditions were set to a pressure difference of 1–4 Pa between the inlet and the outlet orifice to simulate the adjustable oppositely directed convective flow conditions of the TCS. The CFD simulation were performed to assess the flow conditions within the TCS at the orifice between the emission and the detection chamber. The average air velocities were also measured, depending on the pressure difference, using a thermal anemometer (testo 405 i, Testo, Titisee-Neustadt, Germany) at five positions.

The simulated and measured average air velocities resulting from the applied pressure differences were used to investigate the effect of the air velocity on dustiness. For this purpose, the pressure difference between the two chambers was set to values of 0–4 Pa: At a pressure difference of 0 Pa, the plain diffusive transport of ACAM between the detection and the emission chamber was examined, whereas an increase in the pressure difference to 1–4 Pa allowed for the investigation of the diffusive transport of ACAM with an oppositely directed convective airflow. The pressure differences were set by the throttle valve positioned in front of the double-acting ball valve above the detection chamber. The highest achievable pressure difference was 4 Pa. At pressure differences greater than 4 Pa, there is a risk of the bursting of the chambers. The atomization of 100 mg of ACAM was performed as previously described. All measurements were carried out in triplicate.

#### 2.2.5. Determination of the Evacuation Time of the Detection Chamber during the Detection Phase

The quantification of the emission of ACAM dust was enabled by evacuating the detection chamber with an air sampling pump via the IOM sampler equipped with a glass microfiber filter. The determination of the evacuation time is required to ensure a reproducible measurement of the ACAM dust emission within a justifiable time period. For this reason, three different methods were used to determine the evacuation time. One approach was the use of smoke to visualize the flow of airborne particles and their behavior during the evacuation of the detection chamber. Another approach for the determination of the evacuation time was the CFD simulation. A third approach was based on the number of evacuation cycles and thus, the resulting evacuation time necessary for the complete elimination of the dust from the detection chamber.

**Figure 5:**
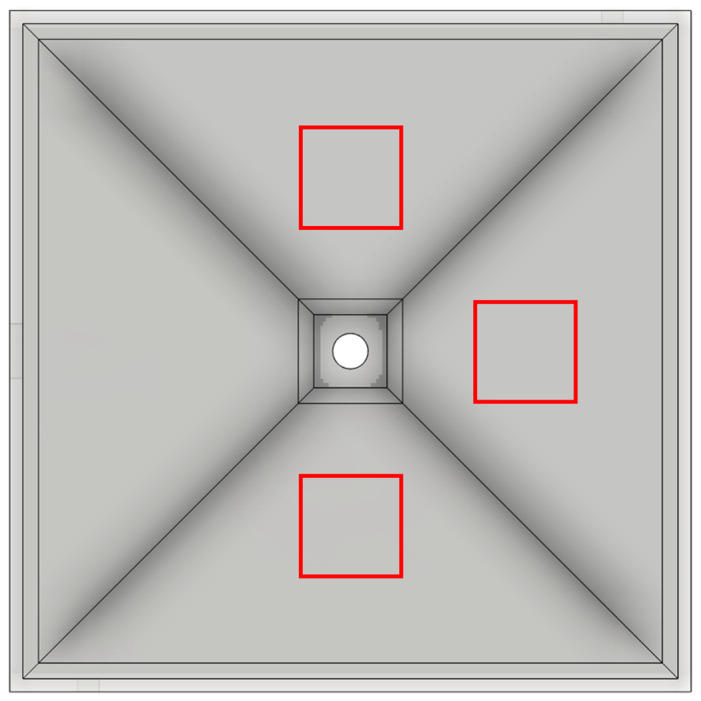
Detection chamber (top view) and the positions of the three square fields (highlighted in red) for the measurement of the mean gray value.

With the first approach to determine the evacuation time, smoke was generated in the detection chamber by using an airflow tester to visualize the flow. The tube end of the airflow tester was connected to the two orifices for 10 s for pressure compensation via the Y-connector to draw the smoke into the detection chamber. A black background was placed below the detection chamber to increase the contrast of the white smoke resulting from a chemical reaction of pyrosulfuric acid present in the filling layer of the airflow tube with the air humidity. The true flow conditions in the detection chamber during each measurement were simulated by using an IOM sampler with a glass microfiber filter. The smoke was evacuated by the air sampling pump, with a flow rate of 5.0 L/min for 15 min. The decrease in the smoke density during the evacuation was monitored using a digital camera (ZV-1, Sony, Tokyo, Japan) mounted centrally above the detection chamber to provide square-shaped pictures. A picture of the white smoke was taken automatically every 5 s. The focus, as well as the ISO value, were adjusted manually to minimize optical disturbances and remained constant during the measurement. In addition, a cold light source (CL 1500 ECO, Zeiss, Jena, Germany) was used to ensure a high level of visibility. The pictures were analyzed with the image processing software ImageJ (US National Institute of Health (NIH), Bethesda, MD, USA) [36] to determine the time point of complete removal of the smoke from the detection chamber. In Figure 5, the top view of the detection chamber is shown with three square fields (200 pixels × 200 pixels) positioned around the center point of the chamber. The mean gray value of each picture, as well as of each resulting square field, was measured. All measurements for determination of the evacuation time were carried out in triplicate.

The second approach to investigate the evacuation time of the detection chamber was the CFD simulation. The same initial conditions were used as in the CFD simulation for the determination of the air velocity of airborne particles in the transport phase of the measurement course (Table 1). The boundary condition was set to a normal velocity (U_n_) of 0.092 m/s at the outlet, which equals the volumetric flow of the evacuated airborne particles. The diffusion coefficient was set to 1 × 10^−8^ m^2^/s in order to identify the zones of airborne particles with a high residence time. Data evaluation of the CFD results was performed with the data analysis and visualization application ParaView (version 5.10.1, Kitware, Clifton Park, NY, USA) [37].

For investigation of the evacuation time in the third approach, ACAM was again atomized in the emission chamber for 5 s, with an overpressure of 50,000 Pa, with the controllable flap closed. Subsequently, the controllable flap was opened for 60 s. Only the plain diffusive transport in the detection chamber was considered. The oppositely directed convective flow was excluded by closing the ball valve located above the detection chamber. Consequently, the pressure difference between both chambers was 0 Pa. To investigate the influence of various evacuation times on the measured dust emission of ACAM, evacuation times of 180, 360, 540, 720, and 900 s were predefined to evacuate the detection chamber up to five times at a flow rate of 5.0 L/min. Furthermore, dust emissions at an evacuation time of 3600 s were measured to investigate whether any significant differences existed in the collected ACAM amounts compared to those collected in the investigations with shorter evacuation times. A time period of 3600 s, corresponding to a 20-fold evacuation of the detection chamber, was assumed to be sufficient for the elimination of most airborne particles from the chamber. All measurements were again performed in triplicate. The amounts of ACAM collected in the glass microfiber filters were quantified by HPLC.

Finally, the three approaches for determination of the evacuation time of the detection chamber were compared (see Section 3.3) to ensure a comparability of the dustiness measurements within a justifiable time period.

### 2.3. Powder Characterization

#### 2.3.1. Bulk Density and Tapped Density

The bulk density and tapped density of ACAM were measured using a jolting volumeter (STAV 2003, J. Engelsmann, Ludwigshafen, Germany) in accordance with the monograph 2.9.34, “Bulk Density and Tapped Density of Powders,” in the European Pharmacopoeia [38]. A 250 mL graduated measuring cylinder was filled with 100 g of ACAM. The V_10_–V_500_ values, the compressibility index (Equation (1)), and the Hausner ratio (Equation (2)) were calculated using the powder density before (bulk density) and after 2500 taps (tapped density):

(1) $$\mathrm{Compressibility}\;\mathrm{index}=100\times \left(\;\frac{\mathrm{Tapped}\;\mathrm{density}\;-\;\mathrm{Bulk}\;\mathrm{density}}{\mathrm{Tapped}\;\mathrm{density}}\;\right)$$

(2) $$\mathrm{Hausner}\;\mathrm{factor}=\frac{\mathrm{Tapped}\;\mathrm{density}}{\mathrm{Bulk}\;\mathrm{density}}$$

The bulk density and tapped density of ACAM were measured in triplicate.

#### 2.3.2. Particle Size Distribution

The particle size distribution of ACAM was investigated by laser diffractometry (Helos KR, Sympatec, Clausthal-Zellerfeld, Germany). A lens with an effective range of 0.5–875.0 µm was used for the measurements, which were performed in triplicate. The ACAM powder was dispersed by using compressed air with an air pressure of 150,000 Pa. The evaluation of the particle size distribution was performed with Paqxos software (Version 2.0.3, Sympatec, Clausthal-Zellerfeld, Germany).

#### 2.3.3. True Density

The true density of ACAM was determined with a helium pycnometer (Pycnomatic ACT EVO, Porotec, Hofheim am Taunus, Germany). Approximately 8.0 g of ACAM were placed in the analysis chamber. The true density value was calculated as the mean of ten measurements. All determinations of true density were performed in triplicate.

#### 2.3.4. Residual Moisture Content

The residual moisture content of ACAM was investigated using thermal gravimetric analysis (TG 209 F1 Libra^®^, Netzsch-Gerätebau, Selb, Germany). The temperature was increased from 25 °C to 160 °C, with a heat rate of 10 K/min. These measurements were also performed in triplicate.

### 2.4. HPLC

The quantification of the collected ACAM in the glass microfiber filter was performed with a VWR-Hitachi Chromaster 5000 (Radnor, IN, USA), equipped with a 250 mm × 4 mm column (LiChroCART^®^ 250-4, Merck, Darmstadt, Germany) containing an RP-18e phase (particle size 5 µm). HPLC measurements were carried out at 22 °C. A mobile phase consisting of a mixture of acetonitrile and water (75:25 *v*/*v*) was adjusted to a pH of 3.5 with phosphoric acid. The flow rate was set to 1 mL/min. A sample volume of 20 µL was injected, and a quantification of ACAM was performed spectrophotometrically at 245 nm. The total run time of one sample was 3.00 min. For elution of ACAM, 1.92 min were required.

The amount of ACAM in each glass microfiber filter was extracted with 2 mL of the mobile phase by shaking with a shaker (Unimax 1010, Heidolph Instruments, Kelheim, Germany) at 75 rpm for 20 min. The concentration of ACAM in the sample solutions was linear in the calibration range between 0.002 µg/mL and 2.212 µg/mL (R^2^ = 0.999).

## 3. Results and Discussion

### 3.1. Validation of the Atomization Process

**Figure 6:**
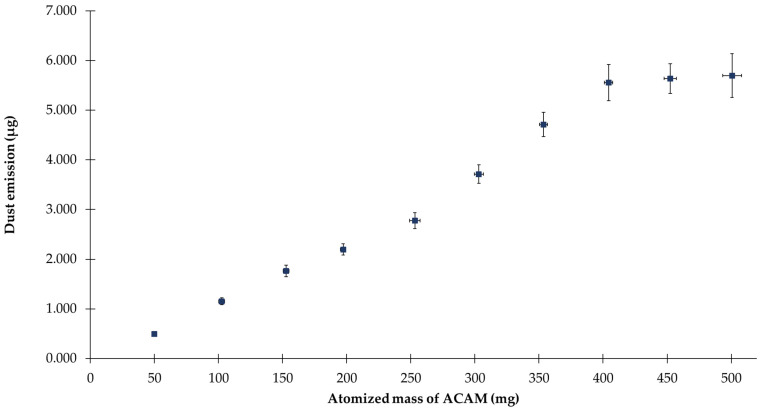
Dust emission versus the atomized mass of ACAM (means ± SD, *n* = 6).

The two-chamber setup (TCS) was developed for the study of the dustiness of pharmaceutical powders by atomizing the powders with an overpressure of 50,000 Pa through the ball valve, centrally located above the emission chamber. The measurement process may be divided into three phases, the first of which is the atomization phase. Accordingly, the purpose of this study was to verify whether a reproducible and precise atomization of pharmaceutical powders is feasible using the TCS. For this purpose, various amounts of the surrogate substance acetaminophen (ACAM) were atomized in 50 mg portions. The dust emission was determined by the IOM sampler mounted in the detection chamber and by the subsequent quantification of ACAM with HPLC. In Figure 6, a linear correlation (R^2^ = 0.9842) between the dust emission and the atomized amount of ACAM can be observed within the range of 50 and 400 mg of atomized ACAM. Within this range, the maximum relative SD is 6.58%. No significant increase in dust emission (*p* > 0.05) was observed within the range of 400 and 500 mg of atomized ACAM. Consequently, a reproducible atomization of ACAM is only possible within the range of 50 and 400 mg.

### 3.2. Results of the Dustiness Measurements during the Transport Phase

The TCS was constructed to study the dustiness of pharmaceutical powders affected by various flow conditions. In this study, four different pressure differences were generated between the emission and the detection chamber. The flows from the emission to the detection chamber resulting from the various pressure differences caused a reduction in the transport of airborne particles and were expected to lower the potential dust transfer from the emission to the detection chamber. The pressure differences between the chambers were obtained by compressed air flowing through the ball valve located above the detection chamber. The pressure difference was maintained for 60 s with the controllable flap open. By varying the pressure differences, resulting in corresponding flows, it was possible to determine whether a relationship exists between the air velocity and the decrease in the transfer of airborne particles from the emission to the detection chamber.

Currently, in the manufacturing of dosage forms containing HPAPIs, pharmaceutical production facilities commonly use negative pressure to reduce exposure due to potential leakage. Therefore, a convective flow from the outside to the inside occurs and prevents this leakage.

**Figure 7:**
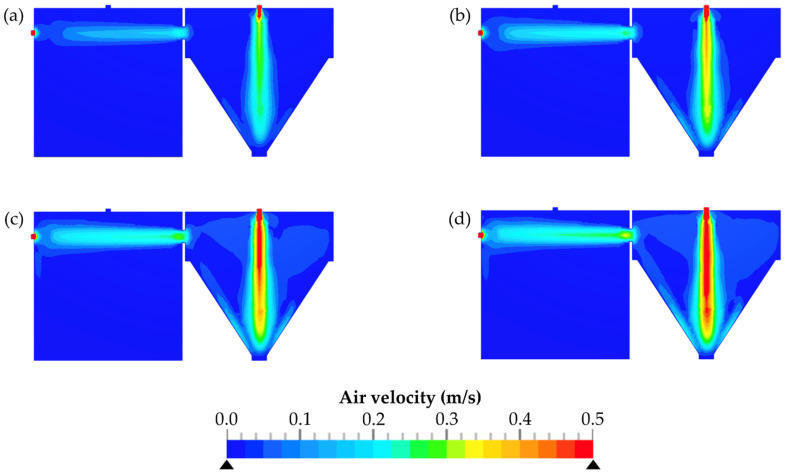
Resulting air velocities depending on the pressure difference between the emission and the detection chamber. Δp values: 1 Pa (**a**); 2 Pa (**b**); 3 Pa (**c**); 4 Pa (**d**).

CFD was used in this study to simulate the resulting flows and flow velocities within the TCS. The air velocity within the setup is particularly crucial at the orifice (diameter 25.4 mm) between the emission and the detection chamber. In Figure 7, the CFD simulations are shown at pressure differences of 1–4 Pa. The four simulations showed that a high air velocity was observed at the position where the compressed air passes the ball valve. It is also apparent that the extent of the airflow affected the average air velocity at the orifice between both chambers, as a function of pressure difference.

The simulated and measured average air velocities are shown in Table 3. Accordingly, generating high pressure differences resulted in higher air velocities between both chambers.

**Figure 8:**
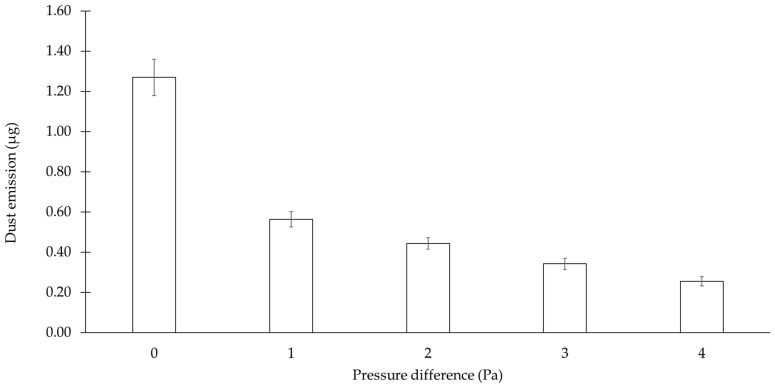
Dust emissions from the plain diffusive transport of ACAM (0 Pa) and from that of ACAM with the oppositely directed convective flow (means ± SD, *n* = 3). For information on the simulated and measured average air velocity, refer to Table 3.

The investigation of the effect of the air flow on dustiness was confirmed by atomizing ACAM, either with 0 Pa, for the examination of the plain diffusive transport of airborne particles, or with 1–4 Pa, for the examination of the diffusive transport with an oppositely directed convective flow. At 1–4 Pa, the oppositely directed convective air flow served as a measure to minimize dust emission. In Figure 8, the resulting dust emissions from various flow conditions are presented. The plain diffusive transport of airborne particles (0 Pa) resulted in the highest dust emission of 1.269 ± 0.091 µg ACAM transported from the emission to the detection chamber for 60 s. A significant decrease in dust emission (*p* < 0.05) was observed during the examination of the diffusive transport of airborne particles with an oppositely directed convective flow. A pressure difference of 1 Pa between the emission and the detection chamber resulted in a significant decrease in the dust emission (*p* < 0.05) to a value of 0.557 ± 0.042 µg ACAM. Furthermore, a significant reduction in dust emission (*p* < 0.05) was observed with increasing the pressure difference between both chambers. Compared to the examination at a pressure difference of 1 Pa, the dust emission was reduced to 0.444 ± 0.029 µg ACAM at a pressure difference of 2 Pa. An increase in the pressure difference to 3 Pa resulted in a further reduction in the dust emission to 0.342 ± 0.027 µg ACAM. The maximum pressure difference of 4 Pa showed the highest reduction in the dust emission to a value of 0.256 ± 0.023 µg ACAM, compared to that of the plain diffusive transport of airborne particles.

The results presented in Figure 8 show that the dust emissions of ACAM resulting from the plain diffusive transport within the TCS may be reduced by an oppositely directed convective flow. Furthermore, the results also indicate that an increase in the pressure difference led to a reduction in the dust emissions. Based on the CFD, the increase in the pressure difference enhanced the airflow velocities at the orifice between the emission and the detection chamber. Consequently, the higher airflow velocities resulted in a decrease in the dust emissions. The above-mentioned investigation showed that the pressure difference set within the TCS as a closed system exerted a substantial effect on the potential transfer of dust.

CFD enables the simulation of the average air velocity depending on the pressure difference between the emission and the detection chamber. These simulated average air velocities appear to be similar to the those found in the measured data, confirming the results of the CFD simulations (Table 3). However, the accuracy of the thermal anemometer is specified by the manufacturer with ±0.1 m/s for air velocities between 0 and 2 m/s. This raises the question of how meaningful the measured values are. In this context, it must be considered that the accuracy increases with declining air velocities and decreases with rising air velocities (for example, the accuracy within an air velocity range of 2–15 m/s is specified with ±0.3 m/s). As the measured average air velocities are very low, with about 0.1 m/s, it is therefore assumed that these values are trustworthy. A further argument for the relevance of the measured average air velocities is their low standard deviation, indicating a high reproducibility.

### 3.3. Determination of the Evacuation Time during the Detection Phase

The detection chamber is an essential part of the TCS, and it has a major impact on the detection phase of the measurement course. Because of the pyramidal design and the diagonal arrangement of the orifices for pressure compensation, most of the airborne ACAM particles should be detected within a reasonable time period. After the transport phase of the measuring course, which lasted for 1 min, the controllable flap was closed to detect the airborne ACAM particles that were transported by plain diffusive transport or by diffusive transport with oppositely directed convective flow from the emission to the detection chamber. For this purpose, the detection chamber containing the air with the airborne particles was evacuated, as previously described. The airborne ACAM particles were retained in the filter and were quantified by HPLC after extraction with the mobile phase.

To ensure that the airborne particles are collected in a reasonable time, CFD, flow visualization using smoke, and dustiness measurements with various evacuation times were performed. CFD was also used to evaluate the airflow within the detection chamber during evacuation.

**Figure 9:**
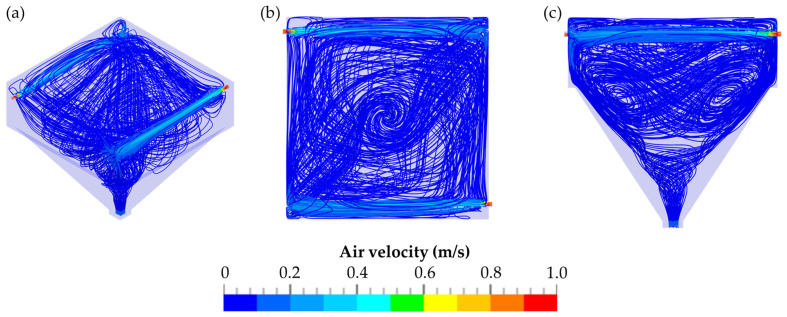
Particle trace analysis of the detection chamber during evacuation: overview of the TCS (**a**); top view of the TCS (**b**); lateral view of the TCS (**c**).

A particle trace analysis was also performed with CFD, and the results are displayed in Figure 9. The negative pressure generated by the air sampling pump was compensated for by incoming air flowing through the diagonally arranged orifices (Figure 9a,b). Initially, the air flows were parallel to the walls of the detection chamber. Because of the diagonal arrangement of the orifices and the impingement of the airflow onto the walls opposite to the orifices, a rotation of the airflow occurred. The rotating airflow also shifted downwards within the detection chamber towards the IOM sampler attached to the bottom of the chamber (Figure 9c). The highest velocity of the airflow was measured directly at the orifices. After the incoming air passed the orifices, the velocity of the airflow within the chamber decreased.

**Figure 10:**
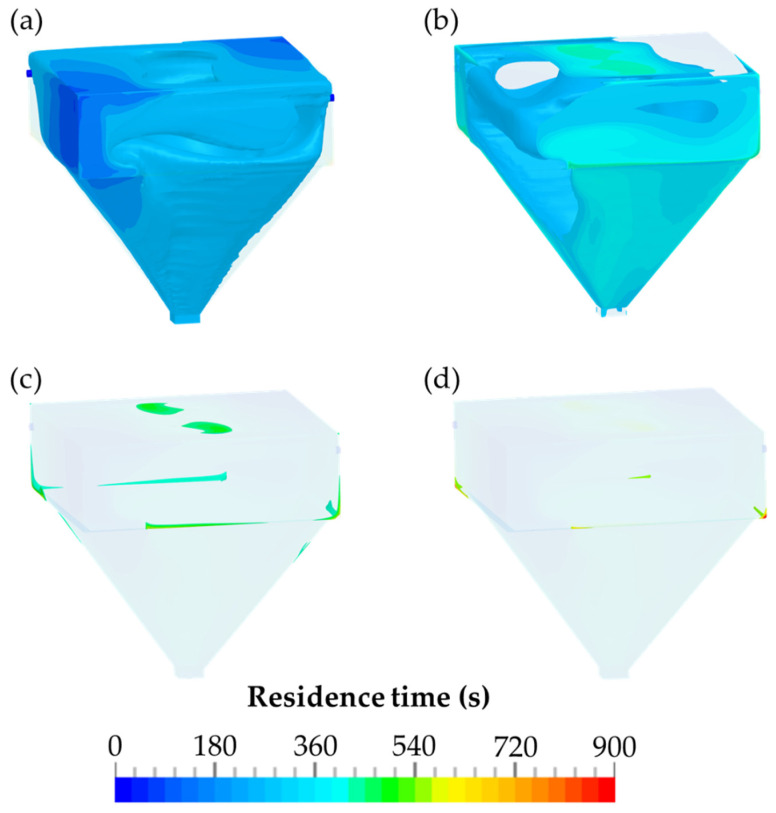
The residence time of the airborne particles within the detection chamber for various time periods: (**a**) 0–180 s; (**b**) 180–360 s; (**c**) 360–540 s; (**d**) 540–900 s.

The residence time of the airborne particles within the detection chamber within different time periods is presented in Figure 10. The zones of the airborne particles, with a residence time between 540 and 900 s, are illustrated in Figure 10d. Compared to Figure 10a–c, which comprised three time periods of 180 s each up to the time point of 540 s, these zones are relatively small. These zones are located exclusively on the walls at the upper part of the detection chamber.

In Figure 10a–c, the time course of the evacuation of the detection chamber is shown in time periods of 180 s. Every 180 s, a 15 L volume was evacuated through the glass microfiber filter of the detection chamber as a result of the flow rate of 5.0 L/min. Simultaneously, 15 L of particle-free air were introduced through the diagonally arranged orifices. The residence times of the airborne particles are shown in Figure 10. They indicate that after a three-fold evacuation of the detection chamber, which takes 540 s, the majority of the particles were already collected by the IOM sampler and could be quantified by HPLC.

The data obtained by CFD was analyzed by the ParaView application, and the total volume of the zones within the different time periods was determined. However, in the simulations, it should be considered that the diffusion coefficient was set to a low value of 1 × 10^−8^ m^2^/s to accurately determine potential zones of airborne particles with a high residence time. These zones are especially perceptible at the transition from the upper part of the detection chamber to the pyramidal part of the chamber. The relative fraction of the zone within the time interval of 0–180 s was 61.92%. After an additioanl 180 s, 99.87% of the airborne particles within the TCS passed through the outlet of the detection chamber. As shown in Figure 10c, the zones for the time interval of 360–540 s are substantially smaller. After an evacuation time of 540 s, a volume of 99.99% was achieved.

**Figure 11:**
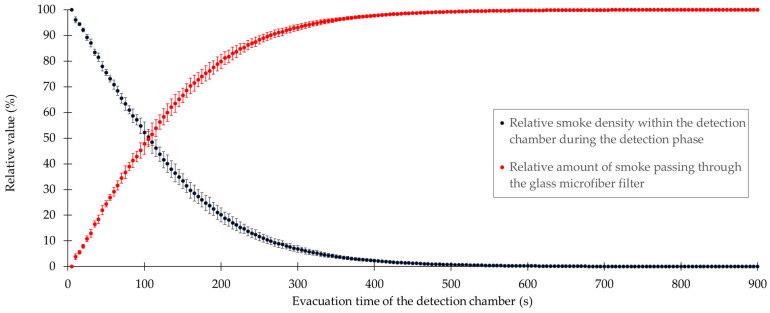
Relative values of the smoke density and the amount of removed smoke versus the evacuation time of the detection chamber.

An additional investigation was performed using smoke to visualize the flow within the detection chamber. For this purpose, smoke was passed through the two orifices for 10 s for pressure compensation to create a homogeneous smoke density. Subsequently, digital camera images were taken while the air sampling pump removed the smoke from the detection chamber at a flow rate of 5.0 L/min. The images were then analyzed for the gray value within the three square fields. The reason for this investigation was to quantify the smoke density and thus, the amount of smoke removed based on the gray value. Therefore, the mean of the gray values of each square field was calculated. The evacuation time-dependent gray values were converted to relative values by defining the highest value as 100% and the lowest value of the resulting exponential decrease at the evacuation time of infinity as 0%. The removal of the smoke from the detection chamber resulted in a decrease in the relative values of the smoke density, as shown in Figure 11. Consequently, the obtained relative values of the exponential decrease in the smoke density were converted into the corresponding values defining the amount of removed smoke.

After determination of the evacuation times of the detection chamber with both CFD simulations and smoke visualizations, additional experiments were performed with the surrogate substance ACAM to investigate whether a significant difference exists between the two evaluation methods. The results of the two evaluation methods revealed that within 900 s a large portion of the airborne particles has already passed through the glass microfiber filter, again in an exponential manner (Figure 11). An exponential decrease in the amount of tracer particles or surrogate substances over time was also observed using other devices for dustiness testing [29]. To ensure that most of the airborne particles were indeed collected within a reasonable time period, the detection chamber was evacuated for 3600 s. The ACAM dust emission measured at this time point was defined as the maximum achievable emission. The results obtained with this evacuation time were compared to those obtained with shorter evacuation times of 180, 360, 540, 720, and 900 s. With the data of the evacuation time-dependent CFD simulations and smoke visualizations, relative values were again calculated for comparative purposes. In Table 4, an overview of the evacuation times determined using CFD, smoke visualization, and dustiness measurements, using ACAM as a surrogate substance, is provided.

For the evacuation time of 180 s, it was observed that the detection of airborne particles was insufficient using all three methods. Moreover, no significant difference (*p* > 0.05) between the relative values obtained by smoke visualization and dustiness measurements was found. However, a significant increase (*p* < 0.05) in the relative values obtained with all three methods was observed by extending the evacuation time to 360 s. The data obtained within the time period of 0–540 s showed that relative values over 99.00% were reached with all three methods. Prolonging the evacuation time to 720 or 900 s did not result in a significant increase (*p* > 0.05) in the relative values. The relatively large deviation of the relative values obtained with CFD from those obtained with the other two methods may likely be explained by the low diffusion coefficient of 1 × 10^−8^ m^2^/s. Therefore, increasing the evacuation time to 720 or 900 s did not result in the detection of higher amounts of dust.

### 3.4. Powder Characterization

As shown in previous studies, the dustiness of powders is influenced by various factors, such as true density, bulk density, and particle size [8,23]. Accordingly, the powder properties of the investigated ACAM were determined. The resulting values for the true density, the bulk density, the tapped density, the residual moisture content, and the particle size, with the respective standard deviations, are listed in Table 5. These values are comparable to other well-known and frequently used APIs.

## 4. Conclusions

The two-chamber setup (TCS) presented in this study is a newly developed containment system created especially for the examination of the dustiness of pharmaceutical powders. A reproducible dustiness investigation of even small amounts of pharmaceutical powders is feasible with this setup, as was shown with the surrogate substance acetaminophen (ACAM). The ACAM used in the dustiness experiments exhibits powder properties comparable to other commonly used pharmaceutical powders. A high reproducibility of the dust emission was observed, as confirmed by low standard deviations and a linear correlation found between the atomized amount of up to 400 mg of ACAM and the resulting dust emissions. The deviation from the linearity for atomized amounts above 400 mg was possibly caused by a saturation effect within the system. Furthermore, the TCS was proven to be suitable for the investigation of the dust emission at different flow conditions (plain diffusive transport and diffusive transport with the oppositely directed convective flow of airborne particles). Particularly, the influence of the oppositely directed convective flow on the dust emissions was investigated, and the results indicate that an increase in the pressure difference between the emission and the detection chamber led to a major reduction in dust emissions. Moreover, a time period of 9 min for the evacuation of the detection chamber was sufficient for the detection of most airborne ACAM particles within the detection chamber. An extension of the evacuation time did not result in a significant increase in the detected particles. Computational fluid dynamics (CFD) simulations revealed that the air flowing through the orifices of the detection chamber for pressure compensation caused a rotating and downward flow towards an IOM sampler. The relative values resulting from the examinations using either atomized ACAM or smoke for the determination of the evacuation time indicate that both methods are comparable. The deviation from the values simulated with CFD may be explained by the low diffusion coefficient of the simulation.

From the results of this study, it may be concluded that the presented TCS allowed for a containment investigation of small amounts of pharmaceutical powders, with regard to their dustiness, within a justifiable time. In future studies, other powder blends will be investigated in regards to their dustiness and their particle flow properties. Moreover, it is of interest to determine whether an additional increase in the pressure difference between the emission and the detection chamber may finally lead to a complete interruption of dust emission.

## Figures and Tables

**Table 1:** Initial conditions of the CFD simulations for the investigation of the transport phase of the measurement course.

Properties	Values
Density	1.196 (kg/m^3^)
Kinematic viscosity	1.529 × 10^−5^ (m^2^/s)

**Table 2:** Airflow properties of the CFD simulations for the investigation of the transport phase of the measurement course.

Properties	Values
Gauge pressure	0 (Pa)
Δp between the inlet and outlet orifice	1, 2, 3, 4 (Pa)
Turbulence kinetic energy [k]	1.297 × 10^−2^ (m^2^/s^2^)
Specific dissipation rate [ω]	12.49 (s^−1^)

**Table 3:** Simulated average velocity and measured average air velocities (means ± SD, *n* = 3) at the orifice between the emission and the detection chamber.

Pressure Difference (Pa)	Simulated Average Air Velocity (m/s)	Measured Average Air Velocities (m/s)
1	0.081	0.08 ± 0.01
2	0.116	0.12 ± 0.01
3	0.144	0.15 ± 0.00
4	0.166	0.18 ± 0.01

**Table 4:** Results of the three investigated methods for the determination of the evacuation time.

Evacuation Time (s)	Numbers of Evacuations	Relative Values ± SD (%)		
		Smoke Visualization	CFD	Dustiness Investigation
180	1	75.24 ± 2.93	61.92	74.00 ± 5.40
360	2	96.50 ± 0.62	99.87	92.14 ± 7.35
540	3	99.51 ± 0.13	99.99	99.18 ± 5.13
720	4	99.93 ± 0.02	100.00	99.47 ± 5.97
900	5	100.00 ± 0.00	100.00	99.56 ± 6.36

**Table 5:** Physicochemical properties of the investigated ACAM (means ± SD, *n* = 3).

Properties	Values
True density	1.2886 ± 0.0024 (g/cm^3^)
Bulk density	0.347 ± 0.003 (g/cm^3^)
Tapped density	0.554 ± 0.008 (g/cm^3^)
Hausner ratio	1.60 ± 0.03
Compressibility index	37.32 ± 1.13 (%)
Residual moisture content	0.138 ± 0.053 (%)
Particle size	
x_10_	1.67 ± 0.08 (µm)
x_50_	8.36 ± 0.30 (µm)
x_90_	24.33 ± 0.90 (µm)

## Data Availability

Not applicable.
